# Bending‐Stable Pressure Sensor Based on Natural Wood Nanofibers for Ultra‐Low Detection Limit

**DOI:** 10.1002/advs.76900

**Published:** 2026-08-07

**Authors:** Xu Zeng, Zhengnan Sun, Yilin Wang, Yao Tan, Ranran Xu, Beomjoon Kim, Juergen Brugger, Xiaosheng Zhang

**Affiliations:** ^1^ School of Integrated Circuit Science and Engineering University of Electronic Science and Technology of China Chengdu China; ^2^ State Key Laboratory of Electronic Thin Films and Integrated Devices University of Electronic Science and Technology of China Chengdu China; ^3^ CREMeB Institute of Industrial Science The University of Tokyo Tokyo Japan; ^4^ Microsystems Laboratory Ecole Polytechnique Fédérale de Lausanne Lausanne Switzerland

**Keywords:** flexible electronics, MEMS （Micro‐Electro‐Mechanical Systems）, nanofibrous membrane, wearable sensors

## Abstract

Flexible pressure sensors, essential for intelligent electronic skins, are valued for their versatility and integration. However, the inherent coupling between the pressure and bending strain effects limits their sensing features atop curved surfaces and hinders ultralow detection thresholds. Inspired by natural nests, we present a bioinspired all‐fiber flexible pressure sensor that effectively decouples pressure and bending strain. Mimicking the interwoven fiber networks, the proposed device employs a fully fiber‐based structure derived from natural wood to replicate the pressure‐bending strain decoupling mechanism. This configuration isolates pressure from strain under bending deformation through synergistic modulation of the dielectric constants and interelectrode spacing. Therefore, it first enables precise pressure detection even on curved and dynamic surfaces. Besides the exceptional conformability and long‐term stability, its detection limit achieves a record of 0.03 Pa. Furthermore, this natural wood‐based sensor demonstrates recyclability through a dissolving‐reproducing strategy and spatial pressure mapping through machine learning, offering a sustainable and cost‐effective solution for physiological and environmental monitoring.

## Graphical abstract

A wood nanofiber‐based flexible pressure sensor with intrinsic bending‐stable performance is developed through a porous electrospun architecture and interdigital electrode design. The device exhibits an ultra‐low detection limit and stable operation under deformation for physiological monitoring and spatial sensing, providing a sustainable strategy for next‐generation wearable electronic skins.

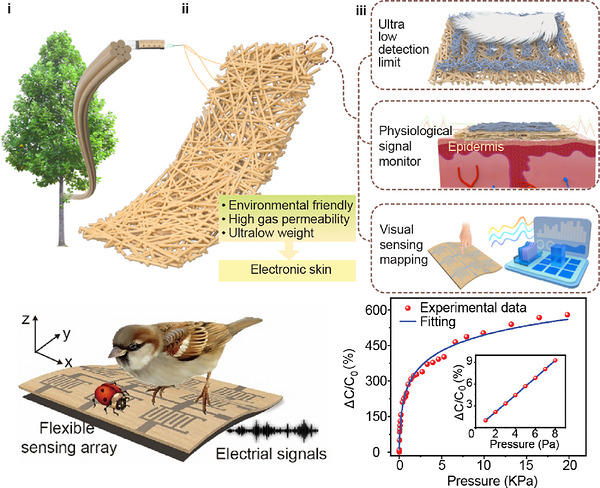

## 1 Introduction

Flexible electronics is an essential emerging research field and is considered the foundation for constructing human‐machine seamless interfaces [1, 2, 3]. These devices are characterized by their lightweight, stretchable, and adaptable, making them ideal for a wide range of applications, including wearable sensors [4, 5, 6], healthcare monitoring [7, 8, 9], soft robotics [10, 11, 12], etc. Among these, flexible pressure sensors have garnered extensive attention owing to their ability to detect physical stimuli such as touch [13, 14], pressure [15, 16], and deformation [17]. However, one of the key challenges in promoting these sensors is the coupling between pressure and bending strain, which complicates the measurement of individual stimuli and limits the sensing performance, especially atop curved surfaces. The interaction of pressure and bending strain is a fundamental issue for flexible pressure sensors, resulting in enormous difficulty in the precise measurement of pressure‐induced signals. Existing strategies for addressing strain interference can generally be classified into two categories. The first involves system‐level calibration under different strain conditions [18], which inevitably increases the overall complexity of the sensing system. The second approach relies on integrating sensing units onto stretchable or highly flexible substrates, thereby transferring the strain‐induced perturbations to the substrate [19, 20]. However, this strategy still fails to fundamentally resolve the intrinsic decoupling issue of individual sensing units. Therefore, achieving effectively pressure‐bending strain decoupling remains a key challenge for next‐generation flexible pressure‐sensing technologies.

Drawing inspiration from nature, particularly the intricate and resilient structures of bird nests, we adopted a bioinspired approach to solve this problem. Bird nests exemplify the effective use of interwoven fiber networks to dissipate stress and maintain structural stability in complex environmental conditions. Supported by current technologies, especially far‐field electrospinning [21, 22, 23], the generated charged jet can form interwoven and stacked fibrous films, akin to the stress‐dissipating structure of bird nests. Unlike dense polymer films that deform uniformly under bending, the interwoven fibers in the lignin membrane can slide, rotate, and rearrange locally. As a result, the bending‐induced strain can be mostly dissipated. From a physical modeling perspective, the inherent self‐adjustment of the effective dielectric constant during the bending process enables the fiber‐stacked substrate to serve as an advantageous structural platform for achieving pressure‐bending strain decoupling.

In addition to advanced processing techniques, the choice of materials is critical for achieving optimal sensor performance. Biomass‐based materials have an edge in next‐generation wearable electronics owing to their natural abundance and sustainability. Among these, lignin, a major component of wood, stands out because of its biocompatibility, biodegradability, and structural versatility [24, 25, 26, 27, 28]. According to *The Global Market for Lignin* report, lignin, traditionally considered a by‐product of industrial processes such as pulping and biorefining, is gaining renewed attention under the global trend toward a sustainable and green economy. Leveraging lignin for wearable electronic devices offers a promising solution that is aligned with the eco‐friendly goals [29, 30, 31].

Herein, we construct a flexible, recyclable, all‐fiber wood‐based pressure sensor using electrospinning technology to endow devices with the ability to decouple pressure and bending strain. The interweaved lignin film, which acts as a functional layer, changes its intrinsic dielectric constant under pressure, enabling highly sensitive and reliable sensing performance. During bending, both the dielectric constant and the interelectrode spacing undergo simultaneous and compensatory changes, effectively minimizing their combined influence on the overall capacitance. This cooperative variation indicates that the sensing behavior is governed by an intrinsic compensation mechanism rather than a passive structural response. Unlike conventional decoupling strategies in flexible capacitive sensors, which typically rely on electrical shielding to suppress external interference or employ signal‐ and algorithm‐based post‐processing to separate coupled outputs without altering the device physics, the present approach achieves decoupling directly at the device level. Specifically, the coupling between dielectric constant and electrode spacing is deliberately engineered to counterbalance under mechanical deformation, thereby stabilizing the overall capacitive response. This intrinsic compensation mechanism provides a fundamentally different design paradigm for achieving pressure–bending strain decoupling within a single sensing unit. Benefiting from this excellent pressure–bending strain decoupling capability, the sensor holds strong potential for applications in wearable electronics and environmental sensing. Furthermore, by integrating machine learning algorithms, the sensor can classify subtle pressure signals generated by various natural stimuli like different bird species, insect movements, and falling leaves. This demonstrates its potential in intelligent, bioinspired sensing systems. Our findings highlight the potential of wood‐based materials as cost‐effective and environmental‐friendly platforms for bioinspired sensing [32, 33, 34, 35, 36].

## 2 Results

### 2.1 Architecture and Designability of the Wood‐Based Pressure Sensor

**FIGURE 1:**
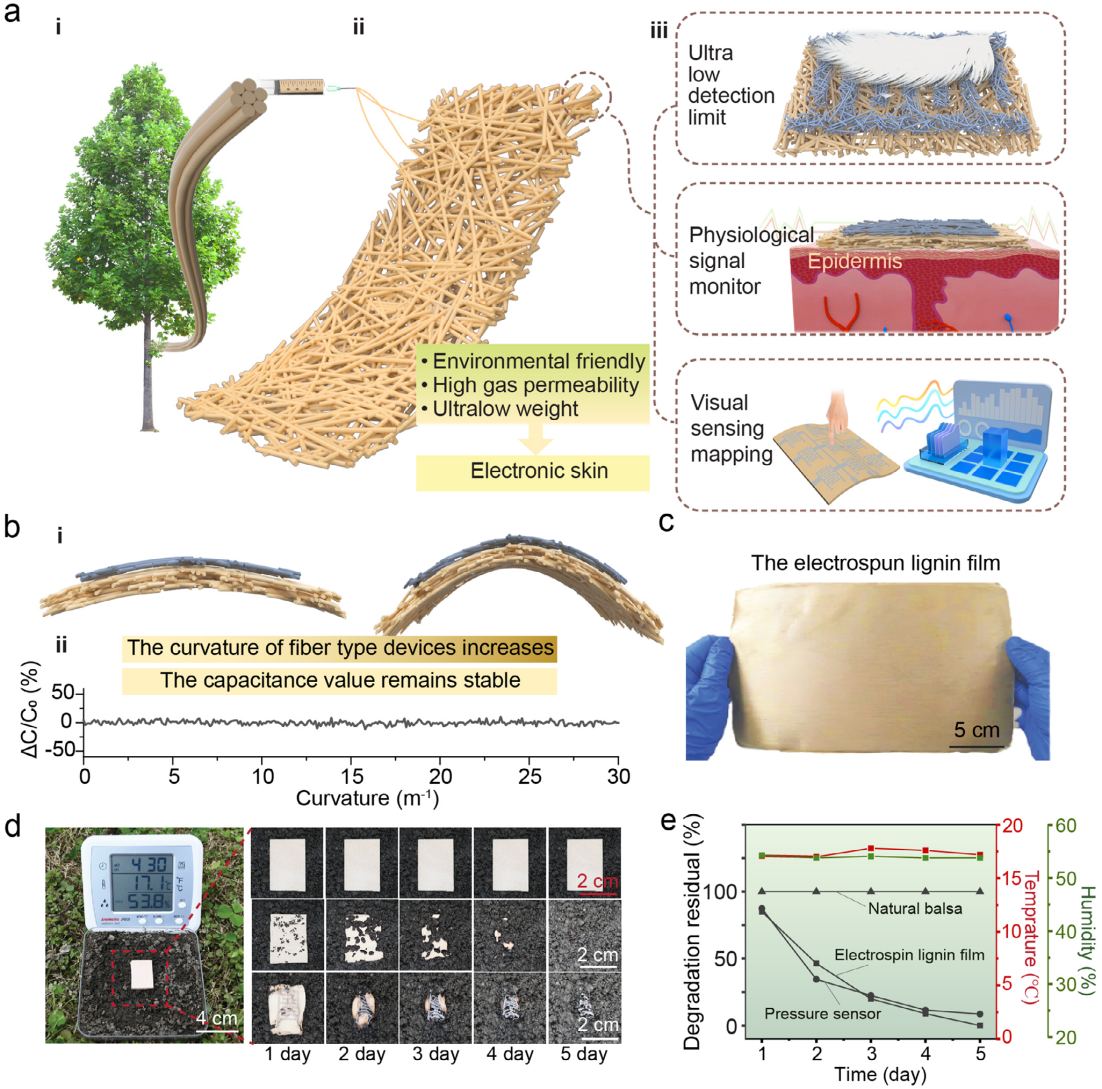
Schematic illustration of the design concept of the wood‐based flexible pressure sensor. (a) (i) fabrication, (ii) structure, and (iii) application of the wood‐based flexible pressure sensor through electrospinning technology. (b) The wood‐based pressure sensor has the property of bending stability. (i) The flexible device has prominent conformability. (ii) The initial capacitance of the device remains stable as the bending curvature increases. (c) Photograph of a large‐area electrospun lignin film. (d) Degradation status of natural Balsa wood, electrospun lignin film, and the capacitive flexible pressure sensor in a natural soil environment. (e) The degradation residual (area retention) of materials over time and the fluctuation of environmental parameters during the degradation process.

The lignin nanofiber membrane was self‐assembled using a far‐field electrospinning apparatus (Figure 1a) with an electrically grounded aluminum foil collector. The specific process is illustrated in Figure S1. The proposed strategy offers exceptional benefits, including environmental sustainability, ultralight weight, and high gas permeability, making it well suited for electronic skin applications. Figure 1b shows a cross‐sectional schematic of the device when subjected to an external force. Notably, the device maintained stable capacitance even when subjected to varying curvatures. The interconnected fibrous network effectively dissipates stress while maintaining mechanical robustness, realizing the decoupling of pressure and bending strain. A large‐area electrospun lignin fiber film (0.15 × 0.24 m^2^) was successfully fabricated via this single‐step self‐assembly (Figure 1c) and remains stable over an extended period under ambient conditions (Figure S2). Based on natural degradation testing conducted under real‐world environmental conditions, the results demonstrate that this device effectively addresses the challenges of biocompatibility and pollution‐free development in wearable applications (Figure 1d,e). The complete degradation process is provided in the Figure S3.

**FIGURE 2:**
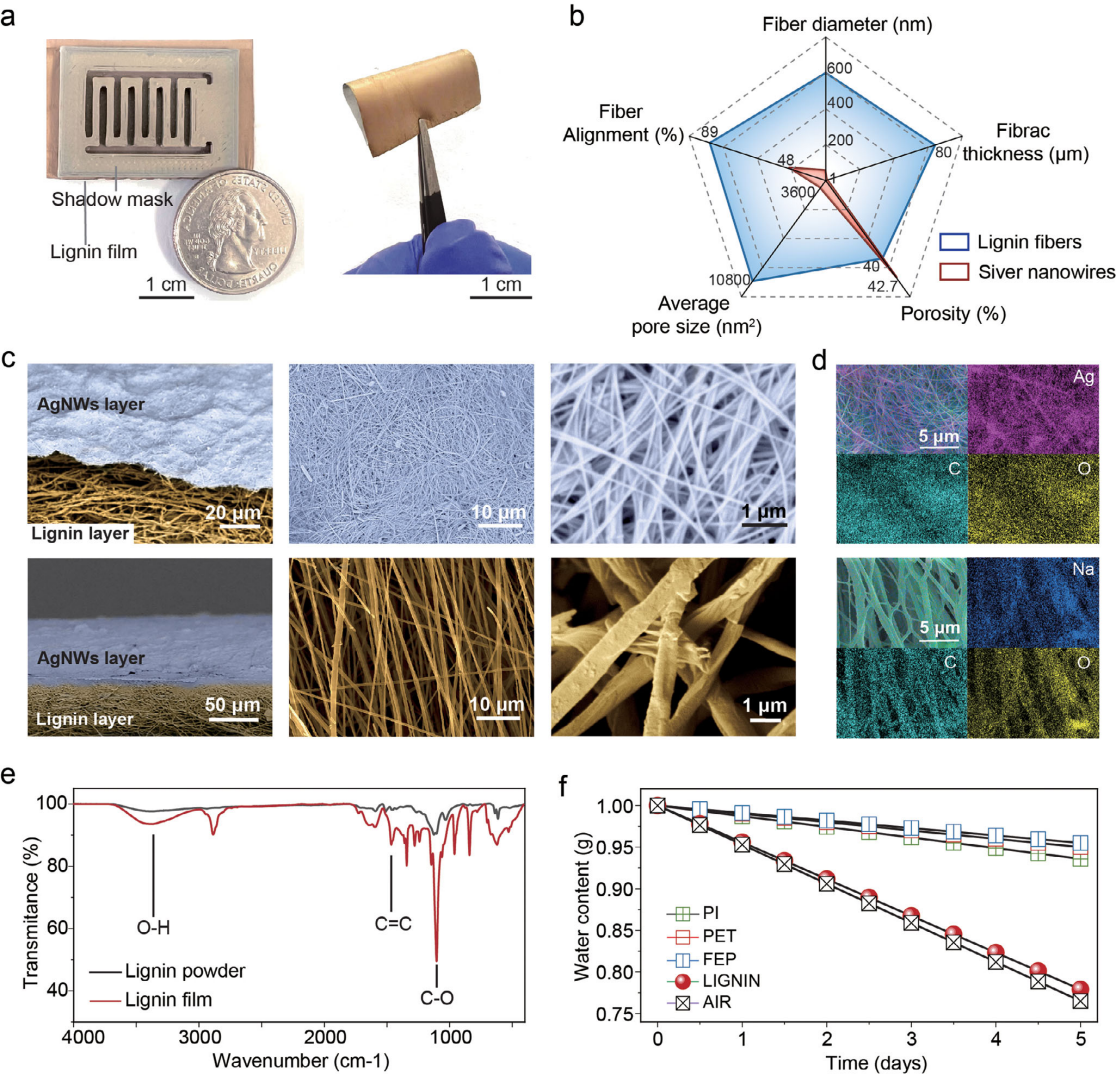
Morphology and microstructure of the flexible and breathable lignin film. (a) Photograph of the fabricated flexible pressure sensor, which is composed of two layers. The lignin film on the bottom layer serves as a support substrate and sensing functional component, and the interdigital electrodes sprayed on it are used for signal output. The flexible lignin film fabricated by the electrospinning process, which can be folded and wound. (b) Radar chart comparing the characteristics of lignin fibers and silver nanowire fibers. (c) SEM images of the device. The electrospun lignin film is covered with a uniform AgNWs layer, and interdigital electrodes have distinct boundary lines. High‐magnification views exhibited many hollows in the AgNWs layer and lignin fiber film, which endows the developed pressure sensor with superior air permeability. (d) EDS scanning images of silver nanowires and lignin fiber film. (e) FTIR spectroscopy of electrospun lignin film and lignin powder. (f) Water vapor transmission tests of three different types of conventional on‐skin film materials and electrospun lignin film.

The obtained pressure sensor and shadow mask template are shown in Figure 2a. The interdigital electrodes have a length of 15 mm, a width of 1.5 mm, and a spacing of 2 mm. The lignin films obtained by electrospinning exhibited good flexibility, allowing them to maintain their structural integrity and stability even after bending, folding, and winding. Its good flexibility is attributed to the all‐fiber structural characteristics of the device (Figure 2b). The specific morphology and structural characteristics are presented in Figure 2c. The interdigital electrodes on the surface were composed of silver nanowires (AgNWs), which were uniformly distributed across the substrate surface, forming a highly conductive network. Importantly, the electrodes retained conductivity even under bending or folding conditions. The electrospun lignin substrate fibers were oriented to a certain degree with an average diameter of approximately 600 nm. Overlapping electrospun fibers form a porous architecture with significant air gaps, which is a critical feature for enhancing both the sensitivity and detection limit of the device at low pressures. Varying the solution ratio resulted in electrospun fibers with distinct morphological structures (Figure S4). The magnified SEM (Scanning Electron Microscopy) images provided further insight into the stacked porous structure of the device. Energy‐dispersive spectrometry (EDS) images showed the uniform distribution of elements in the lignin fibers obtained by electrospinning and silver nanowire electrodes obtained by spray coating (Figure 2d). The FTIR (Fourier Transform Infrared Spectroscopy) spectra revealed that the electrospun lignin nanofibers exhibited more complex and intensified absorption bands, particularly in the C─O, C═C, and O─H regions, than the lignin powder (Figure 2e). These changes indicated molecular rearrangement and enhanced hydrogen bonding during electrospinning (Figure S5). In addition, the ultralightweight nature of the film, with a mass of only 1.4 mg per square centimeter, is attributed to its loose micro/nanofibrous structure, which also imparts excellent air permeability. Water weight loss experiments confirmed that the electrospun lignin film enabled the fastest water vapor transmission compared with conventional flexible on‐skin materials, facilitating skin breathing (Figure 2f). The detailed methods are provided in Figure S6.

### 2.2 Sensing Performance of the Wood‐Based Pressure Sensor

**FIGURE 3:**
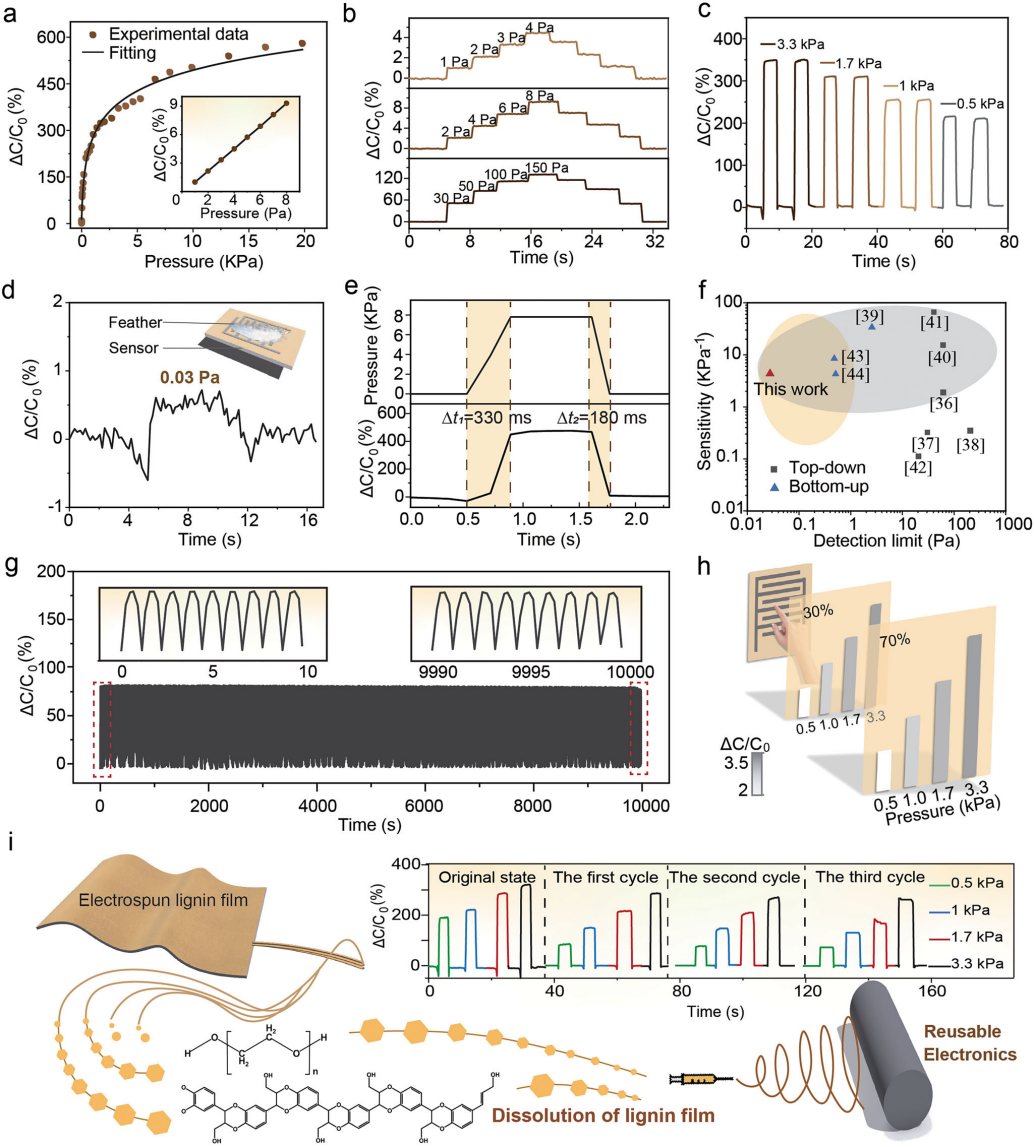
Sensing performance of the wood‐based pressure sensor. (a) Pressure‐response curves for the wood‐based pressure sensors, whose functional layer is the lignin film. The device shows a sensitivity of 4.2 kPa^−1^ in the linear pressure range of 0–500 Pa. (b) The fabricated pressure sensor shows stable and reversible capacitance response under small applied pressures. (c) The device's capacitance change ratio's dependency on the weights of varying masses indicates that the pressure sensing system has excellent reliability. (d) Detection limit of the capacitive wood‐based sensor. The inset shows a feather falling on the wood‐based pressure sensor. (e) The rise and fall time of the capacitance change of the pressure sensor under 7800 Pa. (f) Comparison of sensitivity and detection limit of the wood‐based pressure sensor with those previously reported. The yellow region in the figure represents the low detection limit range, while the gray region indicates the range of high sensitivity. (g) Dynamic capacitance change of the capacitive wood‐based sensor during 10000 cyclic pressure applications. (h) Pressure response of devices under different humidity levels. (i) Performance of pressure sensors prepared in the “dissolving‐reproducing” cycle, which verified the repeatability and recyclability of the process.

Given its unique porous fiber‐like structure and high flexibility, we evaluated the performance of the device for pressure sensing. The sensing characteristics were based on capacitive principles (Figure S7) [37, 38]. Figure 3a illustrates the relationship between the capacitance change ratio and applied pressure. In the range of 0–500 Pa, the sensitivity of the sensor was 4.2 kPa^−1^, which can be considered a linear region. The interwoven nanofibrous porous structure endows the pressure sensor with high sensitivity. However, as the pressure increased, the limited air content within the electrospun fiber film resulted in a gradual decrease in sensitivity, causing the curve to flatten. To investigate the stability and recovery characteristics of the sensor, small weights were successively applied and removed (Figure 3b). Other tests related to small pressure detection, such as rain and flower falling, were explored, with detailed data provided in Figure S8. The pressure sensor device exhibited a stable and repeatable capacitance response under applied pressures ranging from 0.5 to 3.3 kPa, as shown in Figure 3c. After long‐term storage under ambient indoor conditions (4 months), the device maintained stable pressure response performance, as shown in Figure S9. For instance, as shown in Figure 3d, when a feather weighing only 1.8 mg is placed on the pressure sensor, the capacitance increases owing to the compression of the substrate fiber, highlighting the critical role of the unique porous fiber structures in achieving a low detection limit. The capacitance value decreased when the feather was placed and removed, which was attributed to changes in the electric field caused by the proximity of the hand. This ultralightweight sensing is facilitated by the loose and flexible structure of the device, which also enables a rapid response. Figure 3e shows that the response and recovery times of our sensor under an applied pressure of 7800 Pa were 330 and 180 ms, respectively. The dynamic changes at different pressures are demonstrated in Figure S10. The relatively slower response of the sensor arises from inter‐fiber friction and damping within the electrospun lignin network. This trade‐off enhances bending insensitivity, ultra‐low detection limit, and structural stability. Despite the moderate response time, the sensor remains fully suitable for human‐motion and physiological signal monitoring, while future optimization may improve dynamic performance. Figure 3f compares the sensing performance of our wood‐based pressure sensor with that of previously reported wood‐based pressure sensors [39, 40, 41, 42, 43, 44, 45, 46, 47]. Notably, our wood‐based sensor exhibited an optimized pressure‐sensing performance with a sensitivity of 4.2 kPa^−1^ and an ultra‐low detection limit of 0.03 Pa. This superior performance is attributed to the unique fabrication process and the porous flexible structure. In contrast, pressure sensors based on natural wood structures are constrained by a stiff cellulose scaffold, limiting their ability to undergo significant deformation, particularly under subtle pressures. Cyclic loading/unloading tests were conducted to further evaluate the stability of the wood‐based pressure sensor. As shown in Figure 3g, the capacitance profiles exhibited negligible variation even after 10000 loading/unloading cycle tests. The magnified views reveal nearly identical sharp capacitance amplitudes under applied pressures that were well retained over multiple cycles, confirming the high mechanical stability and reliability of the wood‐based pressure sensor for long‐term operation (∼0.02 N). To verify the adhesion robustness between the silver nanowires and the substrate fibers, the surface SEM image after 10 000 cycles is shown in Figure S11. By adjusting the width and spacing of the designed interdigital electrodes, the corresponding pressure response curves are shown in Figure S12. The pressure response performance of the device remained stable under different humidity conditions (Figure 3h). The electrospun lignin film was dissolved in water and subsequently re‐electrospun (Figure S13), highlighting its exceptional recyclability and reusability. Furthermore, the pressure sensor fabricated using the re‐electrospun lignin substrate demonstrated excellent pressure response performance (Figure 3i). The slight decrease in pressure‐sensing performance is associated with the reduced PEO (Polyethylene Oxide) content in the recycled solution, which leads to the formation of beaded fibers in the electrospun network (Figure S14). This structural change deteriorates the uniformity of the fibrous substrate and thus weakens the pressure‐sensing performance. The re‐electrospun lignin was characterized at the molecular level, showing similar chemical properties to the film obtained from the initial electrospinning process (Figure S15). The above pressure testing platform is illustrated in Figure S16.

### 2.3 Mechanism and Simulation of the Decoupling Bending Strain Effect

**FIGURE 4:**
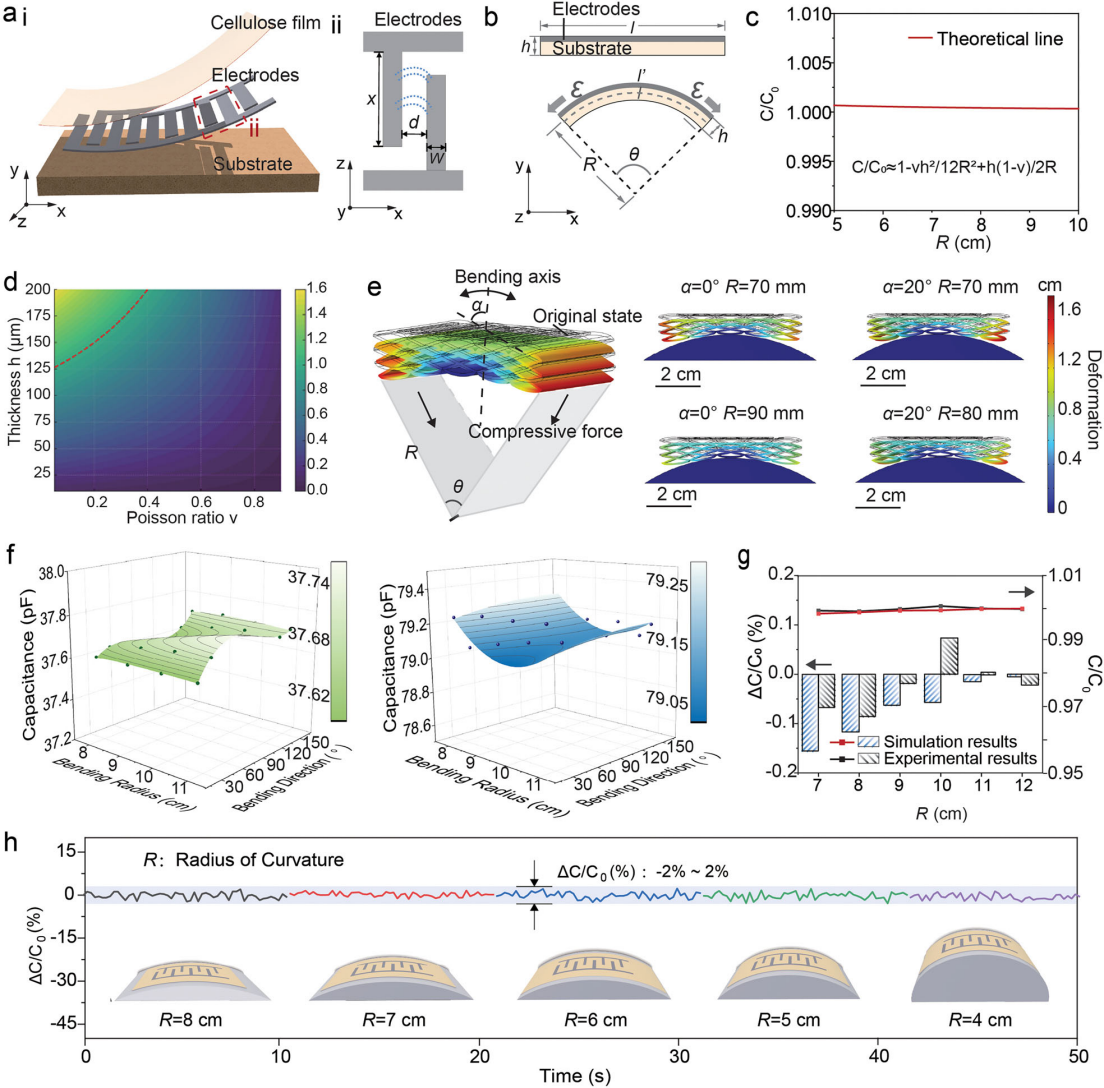
Theoretical analysis and modeling of the proposed configuration. (a) Design of the interdigitated electrode patterns. Simplified schematic diagram of the *x‐z* plane of the device. (b) Simplified schematic diagram of the *x‐y* plane of the device before and after bending. (c) Theoretical curve of the device capacitance as a function of bending radius. (d) Contour map of the deviation ∣C_0_/C_1_‐1∣as a function of Poisson's ratio *v* and film thickness *h*, calculated from the theoretical model. (e) The designed 3D simplified device model in the COMSOL simulation process and the simulation results of the device under different bending conditions. (f) The values and fitting surfaces of the *C_21_* and *C_11_* components of Maxwell capacitance under different bending conditions, demonstrating the bending stability of the device. (g) Simulation and experimental results showing the variation of device capacitance with bending radius. (h) The initial capacitance value of the device remains stable in experiments when attached to surfaces of varying curvature.

When the sensor was attached to surfaces with different curvatures, its initial capacitance remained stable. A simplified structural model was established to quantitatively describe this phenomenon. A coordinate system with the length, thickness, and width directions of the device as the *x*‐, *y*‐, and *z*‐axes, respectively, is shown in Figure 4a,b. The electrospun lignin film has an initial thickness *h* and length *l*. This interdigital pattern on the surface was composed of several electrode fingers. In the interdigital electrode design, two neighboring electrode fingers act as the charging electrodes, whereas the electrospun lignin film in between plays the role of the dielectric layer of a capacitor [37, 48]. Hence, multiple capacitors were connected in parallel to the interdigital capacitive pressure sensor. To represent the bending situation more intuitively, coordinate systems in the *x‐z* and *x‐y* directions are established, which display the design parameters, initial state of the device, and its state during bending. As the resulting film has overall uniformity, the strain‐invariant surface (neutral surface) is located in the middle of the film, and its length remains constant (*l*), the angle at which the film bends is θ, and the corresponding strain is *ε*. Set the initial thickness of the film to *h*, the initial length to *l*, the neutral layer curvature radius to *R*, the bending angle to *θ*, and Poisson's ratio to *ν*.

A coordinate system is established with the *y*‐axis oriented along the thickness direction, where *y* ranges from −*h/2* to *h/2*. In the case of pure bending, the distribution of longitudinal strain along the thickness direction is linear. The normal strain ε_*y*_ represents the variation of the material in the thickness direction. Therefore, after bending, the thickness change of the point originally located at the *y* position is:

$$d\;y^{\prime }=dy\;\left(1+\varepsilon_{y}\right)=dy\left(1-v\frac{y}{R}\right)$$

The cross‐sectional area *A*′ of the bent film is calculated by integration:


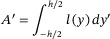




By substituting the formula, we obtain:

$$\;A^{\prime }=\int_{-h/2}^{h/2}\left[\left(R+y\right)\frac{l}{R}\right]\left(1-v\frac{y}{R}\right)dy=lh-\frac{vlh^{3}}{12R^{2}}$$

The dielectric constant ε_r_ of a composite material is determined by the dielectric constant and volume fraction of its constituent materials. In this device, the lignin in the electrospun lignin film was uniformly mixed with air. The air in the electrospun fiber film was expelled under bending deformation. The total volume of the fiber remains unchanged when subjected to bending strain, and the ratio of the dielectric constant can be characterized by the volume of the film before and after bending deformation. By ignoring the variation along the width direction during bending, the ratio of the film volume before and after bending deformation can be replaced by the ratio of the cross‐sectional area before and after bending deformation.

$$\;\frac{\varepsilon_{r_{0}}}{\varepsilon_{r_{1}}}=\frac{lh-\frac{vlh^{3}}{12R^{2}}}{lh}=1-\frac{vh^{2}}{12R^{2}}$$

The actual length ratio after bending on the upper surface is:

$$\;\frac{L^{\prime }}{L}=\left(1+\frac{h}{2R}\right)\;\times \;\left(1-\frac{vh}{2R}\right)=1+\frac{h}{2R}-\frac{vh}{2R}-\frac{vh^{2}}{4R^{2}}$$

Ignore higher‐order terms:

$$\;\frac{L^{\prime }}{L}=1+\frac{h}{2R}\left(1-v\right)$$

Therefore, the ratio of the distance between the interdigital electrodes on the upper surface after bending to before bending is:

$$\;\frac{d^{\prime }}{d}=1+\frac{h}{2R}\left(1-v\right)$$

Here, $\varepsilon_{r_{0}}$ represents the dielectric constant of the film in its initial state, and $\varepsilon_{r_{1}}$ denotes the dielectric constant of the film after bending. The calculation formula for interdigital capacitance is as follows:

$$C=\left(n-1\right)\varepsilon_{0}\;\varepsilon_{r}\frac{S}{d}$$

In this formula, n denotes the number of electrode fingers, ε_0_ is the vacuum permittivity, ε_*r*_ represents the effective relative dielectric constant of the substrate, *S* is the effective overlapping area between adjacent electrode fingers, and *d* is the electrode spacing. The length of the upper surface of the film after bending can be calculated using the arc length formula. So, by using Taylor expansion for the fractional part and ignoring the second‐order small quantity, the interdigital capacitance ratio before and after bending can be described as:

$$\begin{array}{cc} \frac{C_{0}}{C_{1}} & =\frac{\varepsilon_{r_{0}}\cdot d^{\prime }}{\varepsilon_{r_{1}}\cdot d}=\left(1-\frac{vh^{2}}{12R^{2}}\right)\;\times \left[1+\frac{h}{2R}\left(1-v\right)\right]\\ & \approx 1-\frac{vh^{2}}{12R^{2}}+\frac{h}{2R}\left(1-v\right) \end{array}$$

The complete derivation process is provided in the Supporting Information file. The Poisson contraction ratio *v* of the electrospun lignin substrate was 0.92 (Figure S17). For the device used in this experiment, *h* was 75 µm. Substituting this into the above formula shows that the values of the last four terms can be ignored. By using this simplified theoretical model, the interdigital capacitive pressure sensor on a fiber‐type substrate maintains stable initial capacitance for different curvatures and bending angles (Figure 4c). The theoretical model derived from the fiber‐stacked substrate structure demonstrates broad applicability across diverse material systems. This analytical framework provides a rational guideline for designing bending strain‐decoupled pressure sensors, enabling stable and curvature‐independent capacitive responses under various bending conditions.

We sought to verify the feasibility of the above theory through three‐dimensional finite element method (FEM) simulations. Owing to the synergistic influence of multiple parameters, simulating the coupling of multiple physical fields is necessary. Due to the fiber stacking structure of the film obtained by electrospinning, we referred to the structural design of three‐dimensional braided structures for textile composites in the model definition of 3D finite element simulation (Figure S18) [49, 50, 51]. The deviation of the capacitance was calculated based on the theoretical model and is presented in Figure 4d. The simplified model of the device is shown in Figure 4e. The simplified model was adopted to qualitatively illustrate the proposed decoupling mechanism during bending. Non‐uniform strain distribution and fringing electric fields might be considered to make it more suitable for other complex deformation situations. The thin film substrate adopts an angle‐interlocked geometric simplified model using 15 subcells, with an interdigital electrode structure design on the surface, and the center point of the thin film is fixed at the top of the arched support. The angle of the bending direction is α, and the bending radius is *R*. It can be observed that the maximum deformation occurs at the edge of the device. The corresponding internal stress distribution of the device is shown in Figure S19. The simulation results revealed two key phenomena. First, when the film substrate experiences pressure, its thickness decreases due to the compression of air within the fiber network, causing the air to be partially squeezed out. Since the substrate is composed of lignin and air, its equivalent dielectric constant is the result of a comprehensive calculation of the two parts. Thus, bending strain induces changes in the overall equivalent dielectric constant of the thin film. Second, under bending deformation, the film surface stretches, thereby altering the spacing between the interdigital electrodes. The interdigital pattern design of the pressure sensor allows it to have high sensitivity in the *y*‐direction, as small variations in the spacing (*d*) result in detectable changes in the parallel‐connected capacitors. Because these two factors interact, their combined influence must be evaluated through the interaction term rather than as isolated effects. By simulating and calculating the components of the Maxwell capacitance in different directions, we observed that the capacitance values *Cm,_11_* and *Cm,_21_* underwent only negligible changes. When the bending radius and direction were changed, the simulation fitting diagrams of *Cm,_11_* and *Cm,_21_* are shown in Figure 4f. These variations can be ignored when compared with the pressure exerted on the device in the direction perpendicular to its surface. Experimentally, we further measured the initial capacitance under bending along different directions (Figure S20). Figure 4g illustrates the capacitance variation comparison between simulation and experimental results. A statistical analysis of the capacitance output as a function of curvature is provided in Figure S21. As shown in Figure 4h, the device was mounted on an arched mold with various curvatures, and the capacitance value of the device under different bending states was measured using an LCR (Inductance–Capacitance–Resistance) bridge. The results showed minimal fluctuations in capacitance with increasing curvature, with variations attributable to testing deviations. In addition, a parallel‐plate capacitor configuration was employed to measure the capacitance of the dielectric layer, from which the relative permittivity under different bending conditions was calculated, as summarized in Table S2. Furthermore, the long‐term cyclic stability of the device under curved conditions is presented in Figure S22. The electrode maintains stable electrical conductivity under varying curvature (Figure S23). By adjusting the width and spacing of the interdigital electrodes, the device still exhibits bending‐insensitive characteristics (Figure S24). Compared with the identical interdigital electrode structure prepared using flexible polymer film (PDMS (Polydimethylsiloxane), silicone rubber, etc.)(Figure S25), the capacitance exhibited noticeable changes under bending strain (Figure S26). In addition, we verified the same bending‐induced capacitance variation using substrates made from other electrospun materials and randomly porous sponges, as shown in Figure S27. Overall, these results demonstrate that all the evaluated factors had a collaborative impact on the initial capacitance of our electrospun capacitive pressure sensor. Its unique structural design and material parameters ensure that the capacitance of the device remains stable when attached to a curved surface. The pressure sensing performance of the device on different curved surfaces is presented in Figure S28. This study only evaluated the decoupling behavior at a fixed 10 kHz frequency; dielectric dispersion at different frequencies may affect the absolute capacitance values, but the frequency dependence of the normalized capacitance change requires future investigation.

### 2.4 Various Applications of the Wood‐Based Pressure Sensor

**FIGURE 5:**
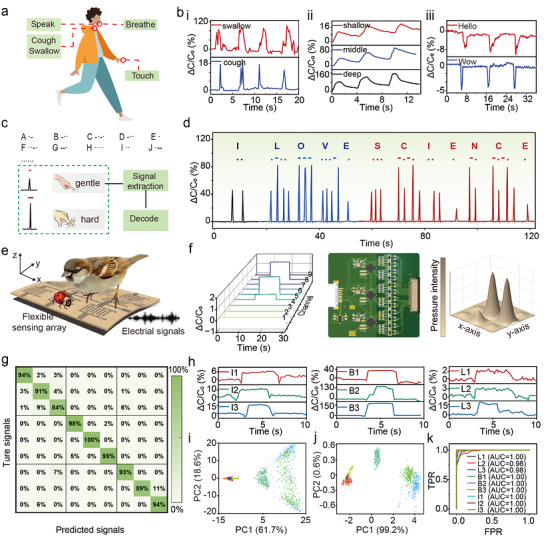
Applications of the wood‐based pressure sensor. (a) The flexible pressure sensor can be applied for human wearable sensing and physiological signal detection. (b)(i) The device can detect cough and swallow signals when attached to the throat. (ii) Comparison of capacitance signal variation under different breathing modes when placing the device under the nose. (iii) Recorded capacitance signals responding to different acoustic words. (c) The Morse code alphabet chart and the flow chart of different touch modes’ signal acquisition and analysis. (d) The Morse code of “I love science” transmitted by tapping the pressure sensor. (e) Scheme diagram for multi‐channel pressure sensor array. (f) Real‐time output capacitance signals when a bird and an insect specimen are placed on the pressure sensor array, and the corresponding signal processing circuit and visualization interface. (g) Confusion matrix results obtained using the SVM algorithm. (h) The dataset comprises nine categories: three differently sized insect species (I1‐I3), three differently sized bird species (B1‐B3), and three types of fallen leaves with varying sizes (L1‐L3). (i) The results of using Principal Component Analysis (PCA) before machine learning training. (j) Comparative analysis of PCA distributions after machine learning training. (k) Receiver operating characteristic (ROC) curves of nine different pressure signals.

Based on the above analysis, our capacitive sensors demonstrated excellent performance and hold significant potential for applications in wearable electronics and related fields [52, 53, 54]. Conventional interdigital capacitive sensors are sensitive to bending strain, leading to varying initial capacitances on curved surfaces [37, 55, 56], which can cause signal detection inaccuracies. Our wood‐based pressure sensor, fabricated via electrospinning, maintained a stable initial capacitance regardless of curvature. In addition, the device exhibited excellent flexibility, allowing it to conformally adhere to curved surfaces, which lays a strong foundation for applications in human wearable sensing and physiological signal detection (Figure 5a). To verify the device's capability in human health monitoring, we evaluated its performance in detecting subtle human motion owing to its high sensitivity. For this purpose, the sensor was attached to the neck skin to monitor muscle movement during coughing. The corresponding mechanical stress characterization is provided in the Figure S29. Owing to its high flexibility, the wood‐based sensor achieved conformal adhesion to the skin, effectively capturing clear physiological signals, as shown in Figure 5b. We tested the swallowing, coughing, and breathing responses. During coughing, rapid contraction of the respiratory muscles produces a sudden outward expansion of the neck skin, leading to a sharp increase in applied pressure and a corresponding capacitance peak. The sensor effectively detected capacitance changes corresponding to different physiological signals, with peak values varying according to the intensity (Figure S30). We further evaluated the capability of our pressure sensor to monitor negligible human motions, considering its high sensitivity. For this purpose, the sensor was attached to the cheek to detect muscle movements during speech. During speech, periodic activation of facial and perioral muscles induces small rhythmic skin deformations, generating subtle pressure variations on the sensor and resulting in the observed distinct capacitance fluctuations. When speaking, the facial muscles contract and exert pressure on the sensor, producing distinct waveforms. Words such as “Wow” and “Hello” generate unique capacitance patterns. Notably, the capacitance decreased during speech, demonstrating the potential of the sensor for acoustic sensing. Additional real‐time physiological signal monitoring results are shown in Figure S31 [14, 57, 58]. For comparison, a PDMS‐based interdigitated capacitive sensor was also employed for physiological signal detection, and the corresponding results are presented in Figure S32.

Beyond physiological monitoring, pressure sensors have also been demonstrated as tools for Morse code‐based communication [59]. A standard Morse code diagram is shown in Figure 5c. Considering the correspondence between pressure signals and capacitance curves, a gentle press represents a “dot”, a hard press represents a “dash”, and each alphabet ends with a several‐second pause. For instance, the sentence “I love science” was encoded using Morse code, with the corresponding capacitive signals shown in Figure 5d. By utilizing this strategy, information transmission can be easily achieved, with capacitive responses for additional words presented in Figure S33. Machine learning techniques can further decode these signals into text to enable efficient communication. This feature is particularly valuable for individuals with severe disabilities, nonverbal patients, or covert communication scenarios, such as military operations.

The function of a single pressure sensor is often limited to localized detection. For spatial pressure mapping, a large‐area pressure sensor array is preferred to mimic the functionality of the human skin. To illustrate this, a 3 × 3 wood‐based flexible pressure sensor array was developed, offering biocompatibility, scalability, and excellent conformability (Figure 5e). When external pressure is applied, the corresponding sensor unit generates a distinct output signal, which is captured in real time using a multichannel data acquisition system. After signal processing, real‐time statistical results can be displayed on the screen [60]. The nine sensing units can display real‐time external pressure intensity (Figure 5f). The relevant backend circuits and visualization programs are shown in Figure S34. When pressing the array, the underlying sensor is compressed, inducing a localized capacitance change. The results of the real‐time change are displayed in the interface of the program, including the capacitance value change of the corresponding sensing unit and pressure intensity visualization. Signal crosstalk between the array units was minimal, with detailed testing methods provided in Figure S35.

The skin‐inspired sensor array demonstrated excellent spatial position recognition but faced challenges in directly distinguishing pressure levels from similar signals. To validate the feasibility of the device operating under natural environmental conditions, machine learning methods have been employed [61] (Figure 5g). Figure 5h shows the response waveforms corresponding to the placement of size‐varied insect specimens (I1‐I3), bird specimens (B1‐B3), and fallen leaves (L1‐L3). Each circumstance was tested 100 times, and a total of 900 capacitance change waveforms were used as the raw information for machine learning. The dataset was split into training and testing sets at a 7:3 ratio and analyzed using a support vector machine (SVM) model [62, 63]. The prediction accuracy of the machine learning model for specific training and testing sets was 92.8%, demonstrating its accuracy, universality, and robustness. Feature extraction was performed to preprocess the data, with key features such as the maximum value, 75th percentile, autocorrelation mean, peak difference, minimum value, variance, mean, and standard deviation selected as feature vectors. Dimensionality reduction was then applied to visualize the data structure in two‐dimensional space while retaining its maximum variability for visualization. Different colored points represent the nine‐group dataset. The samples exhibit poor separation and weak clustering before feature extraction (Figure 5i). After feature extraction, the clear clustering patterns indicate that the data points corresponding to each condition in the feature space are tightly grouped, reflecting the precision of machine learning in distinguishing pressure levels (Figure 5j). Receiver Operating Characteristic (ROC) curves for nine classification categories are presented in Figure 5k. All classes exhibit high classification performance, with Area Under the Curve (AUC) values approaching 1.00, indicating excellent discriminative capability of the SVM classifier. The results demonstrate the feasibility of assembling a large‐area wood‐based flexible pressure‐sensing array for spatial pressure mapping and its potential application value for wearable electronics and electronic skin.

## 3 Discussion

In this study, we developed a biodegradable, recyclable, and highly flexible wood‐based pressure sensor inspired by the interwoven structure of bird nests, fabricated through electrospinning technology. The porous fibrous network not only emulates the mechanical resilience and adaptability of nest architectures but also effectively decouples pressure and bending strain, ensuring accurate pressure sensing even under complex deformations. The resulting sensor exhibited an ultralow detection limit (0.03 Pa), and excellent conformability to human skin, making it suitable for monitoring human motion and physiological signals. Furthermore, a large‐area sensor array was fabricated to demonstrate spatial pressure mapping capabilities. Integration with machine learning allowed the sensor to distinguish between different pressure waveforms, showcasing its potential in dynamic and multipoint sensing applications. This nest‐inspired design, leveraging sustainable wood‐derived materials, offers a biodegradable, recyclable, cost‐effective, and high‐performance solution for applications in personal healthcare monitoring and environmental interaction.

## 4 Methods

### 4.1 Preparation of the Lignin Nanofiber Membrane

The lignin nanofiber membranes were prepared via far‐field electrospinning technology. First, 1 g of lignin powder was dispersed in 30 mL of 0.5 mol/L NaOH aqueous solution and stirred for 30 min. Second, 1 g of PEO powder was gradually added to the above solution and stirred for 30 min until uniform. Next, the precursor solution was placed in a vacuum chamber to remove bubbles at room temperature. The solutions were then filled into a syringe and extruded at a controllable feeding rate of 1 mL/h using an M01‐005 electrospinning equipment (Foshan Lepton Precision M&C Tech Co., Ltd.). The ambient temperature was maintained at 26°C, the rotating cylinder speed was 20 rpm, and the collection material was aluminum foil. The applied positive high‐voltage DC power supply was connected to the needle tip and set to 10 kV, while the collector was grounded. The distance between the needle tip and collector was adjusted to 15 cm. Finally, the lignin membranes on the aluminum foil paper were peeled off and used as the substrate for the pressure sensor. The thickness of the electrospun membrane can be controlled by adjusting the electrospinning time. In this work, a 75 µm‐thick membrane was obtained after 8 h of electrospinning. This air‐permeable fiber film can be used to detect human physiological signals when attached to the surface of human skin and has promising potential applications in the field of smart skin.

### 4.2 Fabrication of the Pressure Sensor

The electrospun lignin film was cut into rectangles with a length of 3 cm and a width of 2 cm. An interdigital electrode shadow mask was prepared using 3D printing for subsequent spray coating. The designed interdigital electrodes consist of four electrode pairs, each with a length of 15 mm, a width of 1.5 mm, and an inter‐electrode spacing of 2 mm. Subsequently, the prepared AgNW solution (4 mg/mL, with an average diameter of approximately 40 nm and a length of 20–60 µm, dispersed in anhydrous ethanol) was added to a spray gun with high pressure for spray coating. The electrospun lignin film was fixed onto a suitably sized glass slide as a substrate for spraying with a shadow mask. After spray coating 30 times and drying for 1 h, a pressure sensor composed of an electrospun lignin film and an AgNW interdigital electrode with a thickness of approximately 1 µm was prepared.

### 4.3 Pressure‐Sensing Measurement Setup

For electrical measurements, the two interdigital electrodes of the device were connected to an LCR meter using wires, and the capacitance was continuously recorded in real time during loading and unloading cycles. The device was covered with a thin paper‐based flexible encapsulation layer. For low‐pressure characterization (<100 Pa), a micro‐pressure loading platform equipped with a calibrated force probe was used to apply precise normal forces to the device. For pressures above 100 Pa, standard calibration masses were employed to generate known pressure levels. By repeatedly applying and releasing the target pressures, the corresponding capacitance variations were measured to evaluate the sensor's pressure response and stability.

## Author Contributions

**Xu Zeng**, **Zhengnan Sun**, **Yao Tan**, **Xiaosheng Zhang** conceived the study. Methodology was developed by Xu Zeng and **Yilin Wang**. The investigation was carried out by Xu Zeng, Tan Yao, **Ranran Xu**, and Xiaosheng Zhang. Visualization was performed by Xu Zeng, Zhengnan Sun, and Yilin Wang. The project was supervised by Xiaosheng Zhang, **Beomjoon Kim**, and **Juergen Brugger**. Xu Zeng prepared the original draft, and Xiaosheng Zhang contributed to the review and editing.

## Conflicts of Interest

The authors declare no conflicts of interest.

## Supporting information




**Supporting File 1**: advs76900‐sup‐0001‐SuppMat.docx.

**Supporting File 2**: advs76900‐sup‐0002‐VideoS1.mp4.

## Data Availability

The data that support the findings of this study are available from the corresponding author upon reasonable request.
