# Supplementary material for: Bending‐Stable Pressure Sensor Based on Natural Wood Nanofibers for Ultra‐Low Detection Limit
Source: Adv Sci (Weinh). 2026 Aug 7;13(59):e76900. doi: 10.1002/advs.76900 (PMC13449535; doi:10.1002/advs.76900)
Supplement: Supplementary file 1 [file ADVS-13-e76900-s001.docx]

**Supporting Information**

**Bending-stable pressure sensor based on natural wood nanofibers for ultra-low detection limit**

Xu Zeng^1,2^, Zhengnan Sun^1^, Yilin Wang^1^, Yao Tan^1^, Ranran Xu^1^, Beomjoon Kim^3^, Juergen Brugger^4^, Xiaosheng Zhang^1,2,*^

^1^School of Integrated Circuit Science and Engineering, University of Electronic Science and Technology of China, Chengdu 611731, China

^2^State Key Laboratory of Electronic Thin Films and Integrated Devices, University of Electronic Science and Technology of China, Chengdu 611731, China

^3^CREMeB, Institute of Industrial Science, The University of Tokyo, Tokyo 153-8505, Japan

^4^Microsystems Laboratory, Ecole Polytechnique Fédérale de Lausanne, Lausanne 1015, Switzerland

**Table of Contents**

[1. Supplementary Methods	4](#_Toc6580)

[1.1 Theoretical derivation of decoupling strain effect	4](#_Toc25770)

[2. Supplementary Figures	13](#_Toc19469)

[2.1 Experimental procedure of preparing electrospun lignin film	13](#_Toc9730)

[2.2 Long-term stability under ambient conditions	14](#_Toc27893)

[2.3 The complete degradation process of electrodes	15](#_Toc18969)

[2.4 Morphology of electrospun lignin fiber film	16](#_Toc19305)

[2.5 FTIR spectroscopy of electrospun lignin fiber film	17](#_Toc12597)

[2.6 Characterization of breathability	18](#_Toc26319)

[2.7 Working mechanism of pressure sensor	19](#_Toc25718)

[2.8 The capacitive response to small pressure	20](#_Toc21966)

[2.9 Stable pressure response of the device after long-term storage	21](#_Toc12747)

[2.10 The dynamics changes at different pressures	22](#_Toc249)

[2.11 Morphology of the sensor after long-term pressing cycles	23](#_Toc20586)

[2.12 Effect of electrode width and spacing on pressure response	24](#_Toc14294)

[2.13 The simplified structure of the electrospun film	25](#_Toc6728)

[2.14 SEM image of the re-electrospun lignin film	26](#_Toc6910)

[2.15 FTIR characterization of re-electrospun lignin film	27](#_Toc2602)

[2.16 Experimental setup for pressure sensing measurements	28](#_Toc27758)

[2.17 Measurement of the substrate Poisson’s ratio	29](#_Toc27448)

[2.18 The simplified structure of the electrospun film	30](#_Toc8009)

[2.19 Simulation results of stress distribution	31](#_Toc20032)

[2.20 Relative capacitance variation as a function of curvature radius under different bending directions	32](#_Toc2696)

[2.21 Statistical analysis of capacitance variation under different curvature radii	33](#_Toc14866)

[2.22 The long-term cyclic stability of the device attached to a curved surface	35](#_Toc32382)

[2.23 Stability of the electrode conductivity under different bending curvatures	36](#_Toc30606)

[2.24 Bending-Insensitive performance under varying electrode dimensions	37](#_Toc15121)

[2.25 Stress–strain curve of the PDMS film	38](#_Toc8034)

[2.26 The variation of capacitance with bending strain for interdigital capacitors based on PDMS substrate	39](#_Toc29969)

[2.27 The variation of capacitance with bending strain for interdigital capacitors based on different porous substrate	40](#_Toc32400)

[2.28 Pressure sensing performance on different curved surfaces	41](#_Toc19235)

[2.29 Stress–strain curve of the electrospun lignin film	42](#_Toc20519)

[2.30 Wearable application scenarios of the pressure sensor	43](#_Toc20241)

[2.31 Wrist waveforms detected by the pressure sensor	44](#_Toc32225)

[2.32 Performance of a PDMS-based interdigitated capacitive sensor under bending conditions	45](#_Toc11899)

[2.33 Morse code-based capacitance waveforms	46](#_Toc26234)

[2.34 Backend program and circuit for visualizing sensor arrays	47](#_Toc7748)

[2.35 Crosstalk testing of array sensors	48](#_Toc24577)

1. Supplementary Methods

# 1.1 Theoretical derivation of decoupling strain effect

**Set parameters**

The initial thickness of the film is h,

The initial length is 𝑙,

Neutral layer curvature radius: R,

Bending angle θ,

Poisson's ratio ν.

$$l=\theta R$$

**The influence of Poisson effect on thickness**

Establish a coordinate system, where y represents the coordinates in the thickness direction, with a range of $y\in\left[ -h/2,h/2 \right]$,

In the case of pure bending, the distribution of longitudinal strain along the thickness direction is:

$$\varepsilon_{x}=\frac{y}{R}$$

Poisson's ratio defines the relationship between normal strain $\varepsilon_{y}$ and longitudinal strain $\varepsilon_{x}$:

$$\varepsilon_{y}=-v\varepsilon_{x}=-v\frac{y}{R}$$

The normal strain $\varepsilon_{y}$ represents the variation of the material in the thickness direction. Therefore, after bending, the thickness change of the point originally located at y position is:

$$dy^{'}=dy\left( 1+\varepsilon_{y} \right)=dy(1-v\frac{y}{R})$$

**Calculate the cross-sectional area after bending**

The cross-sectional area $A'$ of the bent film is calculated by integration:

$$A^{'}=\int_{-h/2}^{h/2} l(y)dy'$$

Length distribution after bending:

$$l\left( y \right)=\left( R+y \right)\theta=(R+y)\frac{l}{R}$$

Micro element variation in thickness direction:

$$dy^{'}=dy(1-v\frac{y}{R})$$

By substituting the formula, we obtain:

$$A^{'}=\int_{-h/2}^{h/2} [(R+y)\frac{l}{R}](1-v\frac{y}{R})dy$$

Open the parentheses and expand the integral:

$$A^{'}=\frac{l}{R}\int_{-h/2}^{h/2} (R+y)(1-v\frac{y}{R})dy$$

$$A^{'}=\frac{l}{R}\int_{-h/2}^{h/2} [R+y-v\frac{y\left( R+y \right)}{R}]dy$$

$$A^{'}=\frac{l}{R}\int_{-h/2}^{h/2} (R+y)dy-v\int_{-h/2}^{h/2} \frac{y(R+y)}{R}dy$$

Calculate the first item:

$$\int_{-h/2}^{h/2} \left( R+y \right)dy=R\int_{-h/2}^{h/2} dy+\int_{-h/2}^{h/2} ydy=Rh+0=Rh$$

Calculate the second item:

$$\int_{-h/2}^{h/2} \frac{y(R+y)}{R}dy=\frac{1}{R}\int_{-h/2}^{h/2} (Ry+y^{2})dy$$

Split calculation:

$$\frac{1}{R}[R\int_{-h/2}^{h/2} ydy+\int_{-h/2}^{h/2} y^{2}dy]$$

The first integral is zero, and the second integral is:

$$\int_{-h/2}^{h/2} y^{2}dy=\frac{h^{3}}{12}$$

Hence,

$$\int_{-h/2}^{h/2} \frac{y(R+y)}{R}dy=\frac{h^{3}}{12}$$

Final cross-sectional area of the electrospun film：

$$A^{'}=\frac{I}{R}[Rh-v\frac{h^{3}}{12R}]$$

$$A^{'}=lh-\frac{vlh^{3}}{12R^{2}}$$

Considering the Poisson effect, the cross-sectional area after bending is slightly smaller than the original area $A=lh$.

The dielectric constant of composite materials is determined by the dielectric constant and volume fraction of their constituent materials. As for this device, the lignin in the electrospun lignin film is uniformly mixed with air. Under pressure, the air in the electrospun fiber film is expelled. The total volume of the fiber does not change when subjected to pressure, the ratio of dielectric constant can be characterized by the volume before and after the pressure of the film.

$$\frac{\varepsilon_{r_{0}}}{\varepsilon_{r_{1}}}=\frac{{V_{fiber}}/{V_{{film}_{0}}}}{{V_{fiber}}/{V_{{film}_{1}}}}=\frac{V_{{film}_{1}}}{V_{{film}_{0}}}$$

Ignoring the variation along the width direction during bending, the ratio of film volume before and after pressure can be replaced by the ratio of cross-sectional area before and after pressure.

$$\frac{\varepsilon_{r_{0}}}{\varepsilon_{r_{1}}}=\frac{A'}{A}=\frac{lh-\frac{vlh^{3}}{12R^{2}}}{lh}=1-\frac{vh^{2}}{12R^{2}}$$

**Calculate the length of the upper surface after bending**

The length of the upper surface of the film after bending is calculated using the arc length formula as follows:

$$L^{'}=(R+\frac{h}{2})\theta$$

The ratio of the length of the upper surface before and after bending:

$$\frac{L'}{L}=\frac{R'\theta}{R\theta}=\frac{(R+h/2)\theta}{R\theta}=1+\frac{h}{2R}$$

Considering the Poisson effect, the strain of the upper surface fibers is:

$$\varepsilon_{x}=\frac{h}{2R}$$

According to the Poisson effect, the lateral strain (perpendicular to the bending direction) is:

$$\varepsilon_{y}=-v\varepsilon_{x}=-v\frac{h}{2R}$$

The actual length ratio after bending on the upper surface is:

$$\frac{L^{'}}{L}=\left( 1+\frac{h}{2R} \right)\times\left( 1-\frac{vh}{2R} \right)=1+\frac{h}{2R}-\frac{vh}{2R}-\frac{vh^{2}}{4R^{2}}$$

Ignore higher-order terms:

$$\frac{L^{'}}{L}=1+\frac{h}{2R}(1-v)$$

Therefore, the ratio of the distance between the interdigital electrodes on the upper surface after bending to before bending is:

$$\frac{d^{'}}{d}=1+\frac{h}{2R}(1-v)$$

The calculation formula for interdigital capacitance is as follows

$$C={(n-1)\varepsilon}_{0}\varepsilon_{r}\frac{S}{d}$$

So, the interdigital capacitance ratio before and after bending can be described as

$$\frac{C_{0}}{C_{1}}=\frac{\varepsilon_{r_{0}}\cdot d'}{\varepsilon_{r_{1}}\cdot d}=(1-\frac{vh^{2}}{12R^{2}})\times[1+\frac{h}{2R}\left( 1-v \right)]$$

Using approximation $\left( 1+a \right)\left( 1+b \right)\approx1+a+b$ (Ignoring the second-order small quantity ab)

$$\frac{C_{0}}{C_{1}}\approx1+\frac{h}{2R}(1-v)-\frac{vh^{2}}{12R^{2}}$$

For the device used in this experiment, and *h_0_* 75 μm. Substituting it into the above formula shows that the values of the last four terms can be ignored.

By substituting different curvature radius into the formula, the calculated results are as follows:

**Supplementary Table 1.** Variation of normalized capacitance with curvature.

| R(mm) | $\frac{C_{0}}{C_{1}}$ |
| --- | --- |
| 50 | 1.00006 |
| 60 | 1.00005 |
| 70 | 1.00004 |
| 80 | 1.00004 |
| 90 | 1.00003 |

**Theoretical derivation considering the angular deviation between the device and bending direction**

Establish a coordinate system, where y represents the coordinates in the thickness direction, with a range of $y\in\left[ -h/2,h/2 \right]$,

In the case of pure bending, the distribution of longitudinal strain along the thickness direction is:

$$\varepsilon_{x}=\frac{y}{R}$$

Poisson's ratio defines the relationship between normal strain $\varepsilon_{y}$ and longitudinal strain $\varepsilon_{x}$:

$$\varepsilon_{y}=-v\varepsilon_{x}=-v\frac{y}{R}$$

When the spacing direction of the interdigital electrodes forms an angle ϕ with the principal bending direction, the effective strain along the electrode-spacing direction can be expressed by strain projection:

$$\varepsilon_{s}=\varepsilon_{x}\cos^{2} \emptyset+\varepsilon_{y}\sin^{2} \emptyset$$

Substituting $\varepsilon_{x}$ and $\varepsilon_{y}$ gives:

$$\varepsilon_{s}=\frac{y}{R}(\cos^{2} \emptyset-\nu\sin^{2} \emptyset)$$

Therefore, the effective length variation along the electrode-spacing direction becomes:

$$l_{\emptyset}(y)=l[1+\frac{y}{R}(\cos^{2} \emptyset-\nu\sin^{2} \emptyset)]$$

Meanwhile, the thickness-direction micro-element variation caused by the Poisson effect is:

$$dy^{'}=dy\left( 1+\varepsilon_{y} \right)=dy(1-v\frac{y}{R})$$

Thus, the effective cross-sectional area after bending is:

$${A_{\emptyset}}^{'}=\int_{-h/2}^{h/2} l_{\emptyset}(y)dy'$$

Substituting the above equations gives:

$${A_{\emptyset}}^{'}=\int_{-h/2}^{h/2} l[1+\frac{y}{R}(\cos^{2} \emptyset-\nu\sin^{2} \emptyset)](1-v\frac{y}{R})dy$$

Expanding the equation:

$${A_{\emptyset}}^{'}=l\int_{-h/2}^{h/2} [1+\frac{y}{R}(\cos^{2} \emptyset-\nu\sin^{2} \emptyset)-v\frac{y}{R}-v\frac{y^{2}}{R^{2}}(\cos^{2} \emptyset-\nu\sin^{2} \emptyset)]dy$$

The first-order terms containing y vanish over the symmetric interval $\left[ -h/2,h/2 \right]$. Therefore:

$${A_{\emptyset}}^{'}=lh-\frac{l\nu}{R^{2}}(\cos^{2} \emptyset-\nu\sin^{2} \emptyset)\int_{-h/2}^{h/2} y^{2}dy$$

$${A_{\emptyset}}^{'}=lh[1-\frac{\nu h^{2}}{12R^{2}}(\cos^{2} \emptyset-\nu\sin^{2} \emptyset)]$$

Assuming that the dielectric constant variation is mainly determined by the volume fraction change of fibers and air, the dielectric constant ratio can be approximated by the cross-sectional area ratio:

$$\frac{\varepsilon_{r_{0}}}{\varepsilon_{r_{1}}}=\frac{A_{\emptyset}^{'}}{A}$$

$$\frac{\varepsilon_{r_{0}}}{\varepsilon_{r_{1}}}=1-\frac{\nu h^{2}}{12R^{2}}(\cos^{2} \emptyset-\nu\sin^{2} \emptyset)$$

For the upper surface of the film, where *y=h/2*, the effective strain along the electrode-spacing direction is:

$$\varepsilon_{s}=\frac{h}{2R}(\cos^{2} \emptyset-\nu\sin^{2} \emptyset)$$

Considering the Poisson-induced shrinkage effect, the spacing ratio of the interdigital electrodes becomes:

$$\frac{d^{'}}{d}=1+\frac{h}{2R}[(1-v)\cos^{2} \emptyset-\nu\sin^{2} \emptyset]$$

The calculation formula for interdigital capacitance is as follows

$$C={(n-1)\varepsilon}_{0}\varepsilon_{r}\frac{S}{d}$$

So, the interdigital capacitance ratio before and after bending can be described as

$$\frac{C_{0}}{C_{1}}=\frac{\varepsilon_{r_{0}}\cdot d'}{\varepsilon_{r_{1}}\cdot d}$$

Substituting the dielectric constant ratio and electrode-spacing ratio gives:

$$\frac{C_{0}}{C_{1}}=[1-\frac{\nu h^{2}}{12R^{2}}(\cos^{2} \emptyset-\nu\sin^{2} \emptyset)]\{1+\frac{h}{2R}[(1-v)\cos^{2} \emptyset-\nu\sin^{2} \emptyset]\}$$

When ϕ=0°, the equation reduces to:

$$\frac{C_{0}}{C_{1}}=(1-\frac{vh^{2}}{12R^{2}})\times[1+\frac{h}{2R}\left( 1-v \right)]$$

which is consistent with the original model. Since h≪R under wearable-scale bending conditions, the angular-dependent capacitance variation remains very small, indicating that the electrospun lignin-based interdigital capacitive sensor can maintain stable initial capacitance under different bending orientations.

2. Supplementary Figures

# 2.1 Experimental procedure of preparing electrospun lignin film

**Supplementary Figure 1.** The preparation process of electrospinning precursor solution. Firstly, prepare sodium hydroxide solution, then dissolve the lignin powder in the alkaline solvent, and gradually add PEO powder to adjust the viscosity of the solution. Finally, place the magnetic rotor and stir the solution evenly. Then add solution to the syringe, and use electrospinning apparatus to fabricate lignin fiber film.

# 2.2 Long-term stability under ambient conditions

**Supplementary Figure 2.** The device was placed on a mechanical arm under ambient conditions, where it remained stable for an extended period.

# 2.3 The complete degradation process of electrodes

**Supplementary Figure 3.** The complete degradation process of electrodes in soil. Photographs taken after 6, 7, 8, and 9 days show the gradual decomposition and disappearance of the electrodes, indicating their good environmental degradability.

# 2.4 Morphology of electrospun lignin fiber film

**Supplementary Figure 4.** (a-c) Scanning electron microscopy (SEM) images of electrospun lignin fibers with thickening agent PEO doses of 0.5g, 1g, and 1.5g, respectively. (d-f) Magnified top-view images of electrospun lignin fibers with thickening agent PEO doses of 0.5g, 1g, and 1.5g, respectively.

# 2.5 FTIR spectroscopy of electrospun lignin fiber film

**Supplementary Figure 5.** FTIR spectroscopy of electrospun lignin fiber film. The stretching vibrations of –OH (hydroxyl) indicate the presence of phenolic and alcoholic hydroxyl groups in lignin, which is consistent with its typical structure. The characteristic aromatic C=C skeletal vibrations further confirm the aromatic structure of lignin.

# 2.6 Characterization of breathability

Deionized water of 1 g was placed in a test tube, and then the electrospun lignin film was attached to the opening of the tube. Other commonly used on-skin flexible materials used for control experiments. All test tubes are placed on a test tube rack in an indoor environment at room temperature and pressure. Meanwhile, the subsequent decrease in weight was tested.

**Supplementary Figure 6.** Gas permeability measurements. Schematic illustrations of test tubes covered by various commonly used on-skin flexible materials.

# 2.7 Working mechanism of pressure sensor

**Supplementary Figure 7.** Schematic diagram of the working mechanism of pressure sensor. When the film is subjected to compressive deformation, the air in the electrospun fiber film is expelled, so the dielectric constant of the capacitor changes, causing a change in the capacitance.

# 2.8 The capacitive response to small pressure

**Supplementary Figure 8.** The capacitive response to the pressure of (a) a carnation petal and (b) a butterfly orchid petal. (c-d) Capacitive pressure response curves generated by water droplets dropped on the device. However, due to the induced charge generated by water droplets falling on the device, the capacitance value will undergo larger changes, so the capacitance change curve cannot reflect the magnitude of pressure.

# 2.9 Stable pressure response of the device after long-term storage

**Supplementary Figure 9.** Stable pressure response of the device after (a) 4 months and (b) 6 months storage under ambient conditions.

# 2.10 The dynamics changes at different pressures

**Supplementary Figure 10.** Transient response and recovery characteristics of the wood-based capacitive pressure sensor under different applied pressures. (a) Dynamic capacitance response at 1 Pa, showing a response time of t₁ = 160 ms and a recovery time of *t₂ =* 320 ms. (b) Dynamic response under 50 Pa, with t₁ = 340 ms and t₂ = 380 ms. (c) Dynamic response under 100 Pa, with t₁ = 330 ms and t₂ = 180 ms. (d) Dynamic response under 150 Pa, with t₁ = 390 ms and t₂ = 320 ms.

# 2.11 Morphology of the sensor after long-term pressing cycles

**Supplementary Figure 11.** SEM images of (a) the electrospun lignin fiber substrate and (b) the interdigital electrodes, respectively, after 10000 loading/unloading cycles. The silver nanowires remained firmly adhered to the electrospun substrate, and the substrate fibers showed no fracture even after long-term cyclic pressure testing.

# 2.12 Effect of electrode width and spacing on pressure response

**Supplementary Figure 12.** Pressure response of sensors with different interdigital electrode widths (w) and spacings (d). The ΔC/C_0_-T curves under varying pressures (0.5-3.3 kPa) show that adjusting the electrode width and spacing does not significantly affect the overall pressure-sensing performance of the device.

# 2.13 The simplified structure of the electrospun film

**Supplementary Figure 13.** The electrospun lignin film was dissolved in water, and the resulting solution was re-electrospun to obtain a new film. The morphology of the lignin films remains consistent after three consecutive “dissolving-electrospining” cycles.

# 2.14 FTIR characterization of re-electrospun lignin film

**Supplementary Figure 14.** SEM image of the re-electrospun lignin film.

# 2.15 FTIR characterization of re-electrospun lignin film

**Supplementary Figure 15.** The re-electrospun lignin film maintains stable chemical properties.

# 2.16 Experimental setup for pressure sensing measurements

**Supplementary Figure 16.** The testing system consists of an LCR meter for capacitance readout, and a micro-pressure control platform for precise pressure application.

# 2.17 Measurement of the substrate Poisson’s ratio

**Supplementary Figure 17.** Measurement of the substrate Poisson’s ratio. (a) Image of the substrate before stretching. (b) Image of the substrate after stretching, where the dashed rectangle represents the original marked region before deformation.

# 2.18 The simplified structure of the electrospun film

**Supplementary Figure 18.** The simplified fiber structure inside electrospun films. When the film is under pressure, the air between the internal fibers will be expelled, and the overall thickness direction will undergo significant changes. This structure refers to the design in the three-dimensional braided structures for the textile composites model. The left image shows the assumed cross-sectional shape of the thin film before being subjected to pressure, while the right image shows the assumed cross-sectional shape of the thin film surface after being subjected to pressure.

# 2.19 Simulation results of stress distribution

**Supplementary Figure 19.** Principal stress vector distribution in the fibrous substrate under applied pressure, simulated using a 3D structural mechanics model in COMSOL. The colored arrows indicate the directions of principal stresses. Red arrows represent regions with high principal stress, while blue arrows indicate areas with low principal stress. The arrow orientation denotes the direction of the principal stresses, and the arrow color reflects the local stress magnitude. The fibrous architecture redistributes stress through fiber sliding and rotation, leading to non-uniform but directionally aligned stress pathways during deformation. It can be observed that the fibrous substrate experiences only small stresses, while the major stress concentration occurs at the interface between the interdigital electrodes and the substrate.

# 2.20 Relative capacitance variation as a function of curvature radius under different bending directions

**Supplementary Figure 20.** (a) The designed 3D simplified device model in the COMSOL simulation process and the simulation results of the device under different bending conditions. (b) Relative capacitance variation (ΔC/C_0_) as a function of curvature radius (R) under different bending directions (α=0°, 45°, and 90°), demonstrating negligible dependence of the initial capacitance on bending orientation.

# 2.21 Statistical analysis of capacitance variation under different curvature radii

**Supplementary Figure 21.** Statistical analysis of capacitance variation under different curvature radii. The normalized capacitance (C/C0, right axis) and relative capacitance change (ΔC/C0, left axis) are plotted as a function of curvature radius (R).

**Supplementary Table 2**. Capacitance and corrected relative permittivity under different bending strains.

| **Radius of curvature (cm)** | **Capacitance (pF)** | **Surface strain** | **Relative permittivity ε_r_** |
| --- | --- | --- | --- |
| ∞ | 32.5979 | 0 | 2.30 |
| 8 | 35.7187 | 0.00625 | 2.50 |
| 7 | 40.1609 | 0.00714 | 2.82 |
| 6 | 43.2832 | 0.00833 | 3.03 |
| 5 | 45.4675 | 0.01000 | 3.18 |

**Measurement method:** The samples were fixed onto cylindrical substrates with controlled radii of curvature (8, 7, 6, and 5 cm), ensuring well-defined bending states. A parallel-plate capacitor configuration was constructed using flexible copper tapes as the top and bottom electrodes, with the electrospun lignin membrane serving as the dielectric layer. The effective device area was maintained at 4×4 cm². The capacitance values were measured using an LCR Meter. Considering that bending may induce thickness reduction due to the compressible porous structure, we further estimated the thickness variation based on the calculated surface strain and a local volume conservation assumption, using d=d_0_(1−ε). The corrected thickness values were then used to recalculate the relative permittivity.

# 2.22 The long-term cyclic stability of the device attached to a curved surface

**Supplementary Figure 22**. The long-term cyclic stability of the device attached to a curved surface with a radius of curvature of 4 cm.

# 2.23 Stability of the electrode conductivity under different bending curvatures

**Supplementary Figure 23.** Resistance stability of the AgNW-based electrodes under different bending curvatures. The resistances between electrodes A1–A2 and B1–B2 remain nearly constant as the bending radius (R) decreases from flat to 4 cm. This stability is attributed to the stacked and interwoven structure of silver nanowires in the interdigitated electrodes, ensuring reliable contact even during bending.

# 2.24 Bending-Insensitive performance under varying electrode dimensions

**Supplementary Figure 24.** Bending-insensitive performance of devices with different interdigital electrode widths (w) and spacings (d). The normalized capacitance remains nearly unchanged as the curvature increase, indicating that variations in electrode width and spacing do not affect the device’s bending insensitivity.

# 2.25 Stress–strain curve of the PDMS film

**Supplementary Figure 25.** Stress–strain curve of the PDMS film under uniaxial tension, showing the mechanical response within the low-strain regime (0–2%). The initial linear region yields an effective Young’s modulus of approximately 11 MPa.

# 2.26 The variation of capacitance with bending strain for interdigital capacitors based on PDMS substrate

**Supplementary Figure 26.** The variation of capacitance value with bending strain for interdigital capacitors based on PDMS substrate. When the PDMS substrate undergoes elastic tensile deformation, the value of the interdigital capacitance monotonically decreases.

# 2.27 The variation of capacitance with bending strain for interdigital capacitors based on different porous substrate

**Supplementary Figure 27.** Capacitance variation of interdigital electrode sensors fabricated on different porous substrates under various bending curvatures. (a) Sensor with an electrospun TPU substrate also exhibits negligible capacitance variations under the same bending conditions. (b) Sensor with a random porous sponge substrate showing significant capacitance changes during bending. These results demonstrate that, unlike random porous substrates, fibrous substrates enable the interdigital capacitor to maintain stable capacitance under bending, confirming the unique bending–strain–decoupling capability of the fiber-based structure.

# 2.28 Pressure sensing performance on different curved surfaces

**Supplementary Figure 28.** The pressure sensing performance of the device on different curved surfaces remains stable. (a) The pressure response of the device on a flat surface is used as a control. (b–f) The pressure responses of the device on curved surfaces with a radius of curvature of 8 cm, 7 cm, 6 cm, 5 cm, and 4 cm, respectively.

# 2.29 Stress–strain curve of the electrospun lignin film

**Supplementary Figure 29.** Stress–strain curve of the electrospun lignin film in the small-strain regime (0–0.1%), showing its compliant mechanical behavior suitable for flexible sensing applications.

# 2.30 Wearable application scenarios of the pressure sensor

**Supplementary Figure 30.** Wearable application scenarios of the pressure sensor. (a) The sensor is attached to the cheek for detecting facial muscle pressure during speaking. (b) The sensor is attached to the neck for sensing swallowing and coughing, demonstrating its potential in wearable human–machine interface applications.

# 2.31 Wrist waveforms detected by the pressure sensor

**Supplementary Figure 31.** Wrist waveforms detected by the pressure sensor.

# 2.32 Performance of a PDMS-based interdigitated capacitive sensor under bending conditions

**Supplementary Figure 32.** Performance of a PDMS-based interdigitated capacitive sensor under bending conditions. (a,b) Capacitance responses during swallowing and coughing when the device is attached to the skin in a bent state, showing significant baseline shift and unstable signal output. (c,d) The same device fails to reliably detect weak voice signals (“Hello” and “Wow”) after conformal attachment, due to deformation-induced capacitance drift and reduced sensitivity.

# 2.33 Morse code-based capacitance waveforms

**Supplementary Figure 33.** The Morse code of “IML” (Abbreviation of Integrated Microsystems Laboratory) is transmitted by tapping the pressure sensor.

# 2.34 Backend program and circuit for visualizing sensor arrays

**Supplementary Figure 34.** Labview program block diagram and PCB circuit board for sensor array visualization

# 2.35 Crosstalk testing of array sensors

Mark each pressure sensing unit of the array as units 1 to 9. To verify the negligible crosstalk signals between different units of the sensor array, first place weights on units 2, 3, 4, 5, 6, 7, 8, and 9 respectively, and record the capacitance changes of unit 1. It can be seen that when other sensing units are subjected to pressure, the curve only shows slight fluctuations. Similarly, by placing weights on units 1, 3, 4, 5, 6, 7, 8, and 9, the crosstalk signal of other units to unit 2 can be recorded, and so on. The corresponding crosstalk curves are shown below.

9

7

6

5

4

8

3

2

1

**Supplementary Figure 35.** When other sensing units are under pressure, the crosstalk signal waveforms of units (a) 1, (b) 2, (c) 3, (d) 4, (e) 5, (f) 6, (g) 7, (h) 8, and (i) 9.
